# Microneedle patch–based delivery of Pyr-Apelin-13 reverses functional and structural deficits in age-related sarcopenia

**DOI:** 10.1038/s41598-026-43238-9

**Published:** 2026-06-08

**Authors:** Chrysoula Kokotidou, Konstantinos Tzortzakis, Jake Lombardo, Hojatollah Rezaei Nejad

**Affiliations:** 1 Anodyne Nanotech, Boston, MA USA; 2 Anodyne Biotech, Athens, Greece

**Keywords:** Sarcopenia, Apelin-13, Microneedles, Transdermal, Muscle function, Mitochondria, Diseases, Drug discovery, Medical research

## Abstract

Sarcopenia, the age-associated loss of skeletal muscle mass and function, currently lacks effective pharmacological interventions. Pyr-Apelin-13, a potent endogenous peptide that stimulates mitochondrial biogenesis and myofiber regeneration, is limited by rapid plasma clearance and the need for frequent injections. We report the first preclinical evaluation of a transdermal Pyr-Apelin-13 microneedle (MN) patch (HeroPatch) in aged mice. The solid-state “drug-in-resin” design achieved near-complete release efficiency (*≈*100% *in vitro*; 83–99% *in vivo*), enabling reproducible multi-milligram delivery with minimal residual loss. Daily MN dosing significantly improved muscle fiber cross-sectional area and grip strength relative to controls, with outcomes comparable to daily intraperitoneal injection. Histological analysis revealed a shift toward larger fiber sizes, consistent with enhanced myofiber remodeling, and mitochondrial DNA content increased concordantly. Weekly dosing produced smaller, non-significant trends, reflecting lower cumulative exposure. These findings demonstrate that HeroPatch enables efficient, reproducible, and non-invasive systemic delivery of Apelin-13, providing a scalable platform for peptide-based therapy of sarcopenia and related muscle-wasting disorders.

## Introduction

Sarcopenia—the progressive loss of skeletal muscle mass, strength, and function with age—is a major contributor to frailty, falls, and morbidity in older adults. Despite its growing prevalence and clinical impact, *there are no United States Food and Drug Administration (FDA)–approved drugs for sarcopenia as of 2024*. Current management relies primarily on resistance exercise and nutritional support, which are variably accessible and often insufficient in advanced disease^2,3^. Recent regulatory activity underscores the unmet need (e.g., FDA Fast Track designation for LPCN-1148 in a cirrhosis-associated sarcopenia setting), but no therapy has yet achieved approval for primary age-related sarcopenia^4^.

Among emerging biologic candidates, the peptide hormone **apelin** and its receptor **APJ** (APLNR) have gained attention as key regulators of skeletal muscle metabolism, regeneration, and mitochondrial homeostasis. In a landmark study in *Nature Medicine*, Vinel *et al.* demonstrated that apelin production declines with age in humans and mice, and that restoring apelin signaling—through daily pyroglutamate apelin-13 administration or muscle-targeted gene delivery—reversed age-associated sarcopenia by improving grip strength, fiber cross-sectional area, and mitochondrial biogenesis^1^. Additional studies have since expanded apelin’s known benefits: chronic apelin treatment enhances mitochondrial oxidative capacity and fatty acid oxidation in insulin-resistant muscle^8^, increases glucose utilization and insulin sensitivity^9^, activates the AMPK–PGC-1*α* pathway to prevent atrophy in kidney disease models^10^, and promotes angiogenesis and muscle regeneration through vascular niche activation^11^. Collectively, this literature identifies apelin as a potent “exerkine” and therapeutic candidate for muscle-wasting conditions.

However, apelin’s translation to clinical use has been constrained by two major challenges: (1) **its short plasma half-life**, on the order of minutes, which necessitates continuous infusion or frequent injection to maintain efficacy, and (2) **the absence of a patient-friendly delivery route**. While infusion pumps can sustain therapeutic exposure, they are impractical for chronic outpatient use. Modified apelin analogs such as LIT01-196 (a metabolically stabilized apelin-17 derivative) have been developed to extend half-life^12^, but chemical stabilization may alter receptor signaling bias or pharmacodynamics. These limitations underscore the need for an alternative formulation and delivery technology that can provide sustained bioavailability of the native peptide.

Transdermal **microneedle (MN)** technologies provide a promising solution. MNs enable minimally invasive delivery of peptides and biologics while bypassing the stratum corneum and first-pass metabolism^5,6,13,14^. Among MN formats, solid-state “drug-in-resin” or *macronido*-type microneedles offer unique advantages: the peptide is incorporated as a dry powder directly into a rigid, water-permeable scaffold, eliminating solvent exposure, preserving peptide stability, and supporting milligram-level payloads. Release can be tuned by particle size and excipient composition, allowing sustained diffusion from microchannels created by microneedle insertion.

**Fig. 1:**
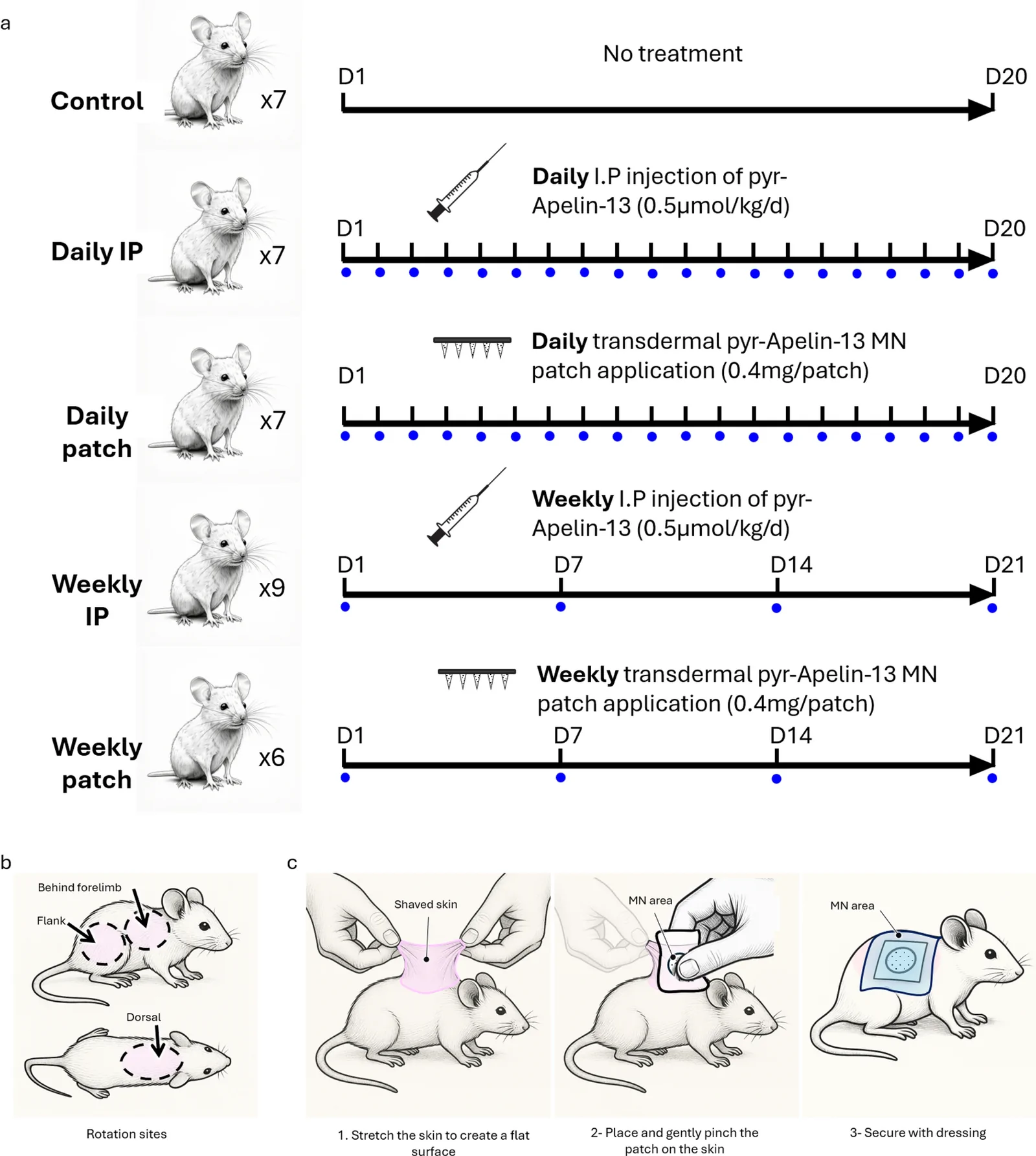
Study design and microneedle (MN) patch application procedure. (**a**) Experimental groups and dosing regimens over 20–21 days: Control (no treatment), daily intraperitoneal (IP) injection of Pyr-Apelin-13 (0.5 *μ*mol/kg/day), daily transdermal Pyr-Apelin-13 MN patch application (0.4 mg/patch), weekly IP injection (0.5 *μ*mol/kg), and weekly MN patch application (0.4 mg/patch). Blue dots mark treatment days. (**b**) Rotation scheme for MN application sites, alternating between dorsal, flank, and behind forelimb regions to minimize localized skin irritation. (**c**) Application procedure: shaved skin was stretched to create a flat surface, patches were gently pressed to ensure microneedle insertion, and a light dressing was applied to secure the patch during wear.

Our previous work has demonstrated the feasibility of this approach for small molecules and biologics using a polymeric porous microneedle platform^7^. In the present study, we extend this technology to deliver **Pyr-Apelin-13** transdermally, testing whether daily application can reproduce the functional, structural, and mitochondrial benefits previously achieved only with daily intraperitoneal injection. We compare no treatment, daily IP, daily MN, and weekly MN regimens in aged mice across grip strength, fiber morphology, and mitochondrial DNA endpoints, while also quantifying delivery efficiency and patch performance (Fig. 1). We hypothesize that sustained release from a solid-state MN patch can provide a non-invasive, patient-compatible route to harness Apelin’s full therapeutic potential for combating age-related sarcopenia.

## Materials and methods

### Mechanism and engineering of the HeroPatch platform

HeroPatch is a non-dissolving, solid-state “drug-in-resin” microneedle (MN) platform engineered to decouple mechanical penetration from drug release. Unlike dissolving MN systems that deposit polymer into tissue and are limited by dissolution kinetics, HeroPatch MNs are fabricated from a rigid, water-permeable resin that functions solely as a structural scaffold: the resin does not dissolve or fragment in tissue and is removed intact after wear. Peptide payloads are incorporated as dry microparticles that are embedded directly into the resin matrix using a solvent-free, dry manufacturing process. This approach (i) eliminates residual solvent concerns, (ii) preserves labile peptide activity, and (iii) supports room-temperature formulation stability.

**Fig. 2:**
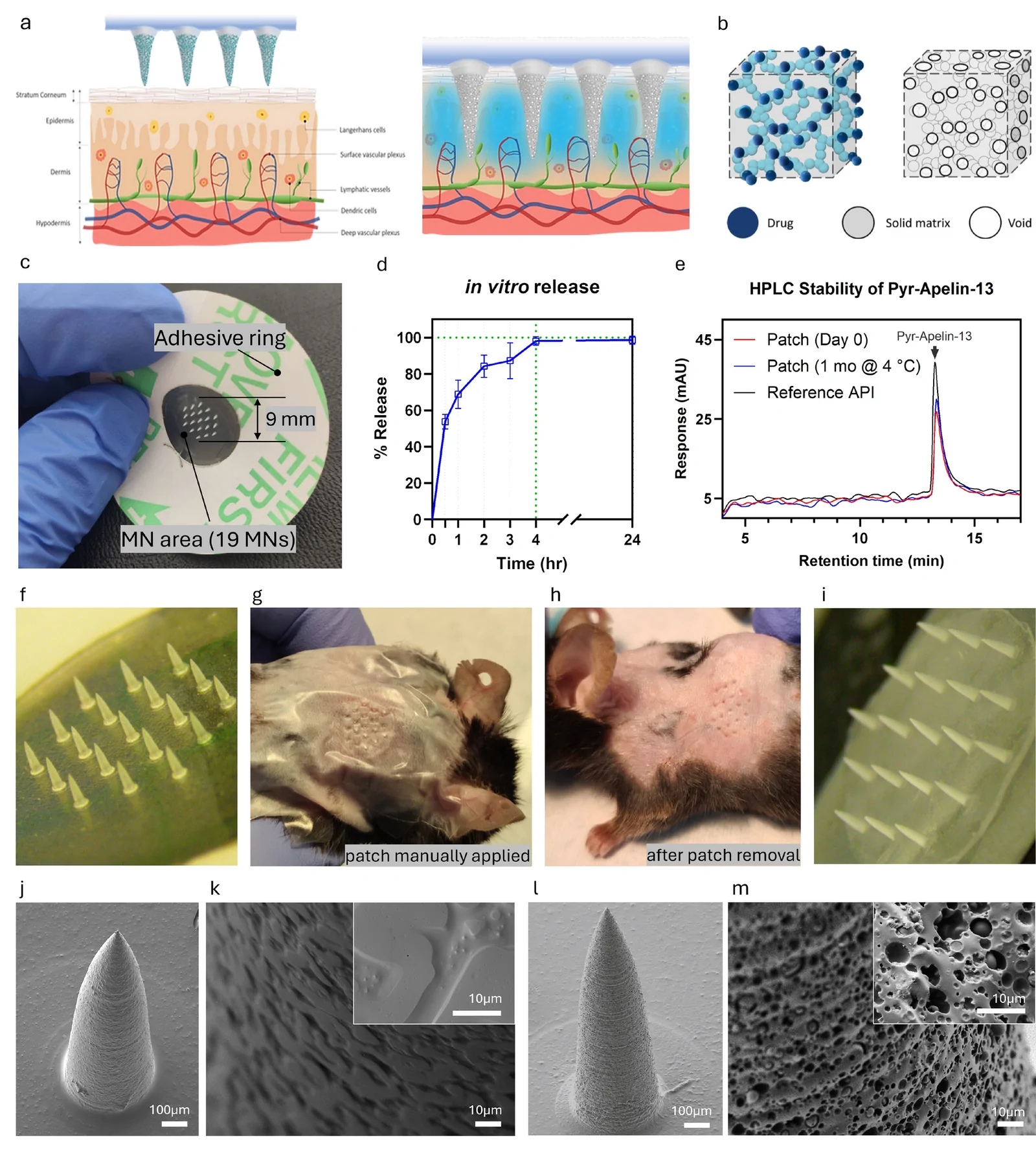
HeroPatch: mechanism and validation. (**a**–**b**) Schematic of the drug-in-resin MN mechanism (dry drug microparticles embedded in a rigid, water-permeable resin) and a conceptual microstructure before/after release. (**c**) Patch used in this study (19 MNs; active area *≈*9 mm). (**d**) *In vitro* Pyr-Apelin-13 release (mean ± SD): *∼*100% release by 4 h. (**e**) HPLC overlays (reference API, day-0, 1 month at $4\,^\circ \textrm{C}$) show no degradants. (**f**–**i**) Optical images: intact MNs, in vivo application, puncture array after removal, and removed MNs showing whitening/porosity consistent with payload depletion. (**j**–**m**) SEMs pre- and post-release show transition from a dense, non-porous surface to an open, porous scaffold after drug egress (scale bars as indicated). Patches were applied manually to shaved, stretched dorsal skin by bilateral pinch/press (10–30 s); removal produced discrete puncture marks without visible erythema, and SEM/optical morphology supports the proposed fluid-mediated dissolution mechanism. See Methods and^7^.

Dose is controlled by the pre-measured dry drug mass embedded into each needle rather than by variable polymer dissolution, improving dose reproducibility and batch-to-batch accuracy. Upon insertion, interstitial fluid wicks into the needle surface, dissolves the peptide microparticles at the needle–fluid interface, and enables diffusion of active peptide through microchannels into dermal tissue while the inert resin scaffold remains. (see Fig. 2a–b) Because drug loading uses the solid (dry) form of the active, loading densities of 1.0–1.5 mg cm^−2^ are achievable (formulation dependent), enabling therapeutic-level peptide dosing that is typically out of reach for conventional dissolving MNs (commonly limited to 0.05–0.15 mg cm^−2^).

This strict separation of structural and release functions addresses two common translational bottlenecks: reliable, reproducible dosing and a manufacturing pathway compatible with high-volume GMP production. Fabrication and analytical characterization follow standardized polymeric MN workflows and are described in Methods (and in related work, e.g^7^).

### Materials

Pyr-Apelin-13 (pyroglutamate apelin-13) peptide was purchased from Sagechem with a reported purity of >99.1%, as confirmed by the manufacturer’s certificate of analysis. The peptide was supplied as a lyophilized powder and stored at $-20~^\circ \textrm{C}$ until use. For intraperitoneal administration, Pyr-Apelin-13 was reconstituted in sterile phosphate-buffered saline (PBS). For microneedle fabrication, the peptide was incorporated in solid-state form directly into the microneedle matrix.

Microneedle patches (HeroPatch) were fabricated using a biocompatible, medical-grade, photocurable resin as the structural matrix material. Tween-80 and sodium phosphate dibasic dihydrate (Sigma-Aldrich) were used as excipients. Silicone microneedle molds were employed to form the microneedle arrays prior to curing.

For high-performance liquid chromatography (HPLC) analysis, trifluoroacetic acid (TFA) was obtained from Carl Roth, and acetonitrile (HPLC grade) was purchased from Sigma-Aldrich. Chromatographic separation was performed using a reverse-phase C18 column (5 µm particle size, Phenomenex) on an Agilent 1260 Infinity II HPLC system. Phosphate-buffered saline (PBS; Sigma-Aldrich) was used for in vitro release studies, residual drug extraction, and skin swab analysis.

Genomic DNA, including nuclear and mitochondrial DNA, was isolated from skeletal muscle samples using the Mag-Bind Blood & Tissue DNA HDQ 96 Kit (Omega Bio-Tek, Catalog No. M6399) according to the manufacturer’s instructions, with automated extraction performed on a KingFisher Apex Automated Extraction System (Thermo Scientific, Catalog No. 5400930). Quantitative polymerase chain reaction (qPCR) was performed using the KAPA SYBR FAST qPCR Master Mix (2*×*) Kit (KK4602, KAPA Biosystems). Each 10 µL reaction contained 5 µL of master mix and was run on a QuantStudio 5 Real-Time PCR System (Thermo Scientific, Catalog No. A28569).

### Study overview and compliance

This study was designed as a controlled, preclinical proof-of-concept investigation to evaluate the feasibility and biological effects of transdermal Pyr-Apelin-13 delivery using a solid-state microneedle patch in aged mice. Functional, histological, and molecular endpoints were assessed to examine treatment-associated changes in muscle performance, muscle fiber morphology, and mitochondrial DNA abundance over a 20-day intervention period.

Twenty-month-old male C57BL/6 mice were randomly assigned to one of five experimental groups: untreated control, daily intraperitoneal Pyr-Apelin-13, daily microneedle patch delivery, weekly intraperitoneal Pyr-Apelin-13, or weekly microneedle patch delivery. Group allocation was performed following acclimation to ensure comparable baseline characteristics across groups.

All animal experiments were conducted at ALTP and were approved by the ALTP Protocol Evaluation Committee, in accordance with applicable national and European legislation on the protection of animals used for scientific purposes (Directive 2010/63/EU and Presidential Decree 56/2013), under authorization number 454824/14-04-25. All procedures complied with institutional animal welfare policies and relevant regulatory requirements. Procedures were performed under appropriate anesthesia where applicable, and all efforts were made to minimize animal distress.

### Animal model and study design

**Study type.** Randomized, controlled, parallel-group in vivo study with blinded operators and analysts; single site.

**Study dates.** Conducted from **2025-03-26** to **2025-04-15**; daily functional measurements were collected on Days 1–20, and terminal tissue collection occurred on Day 20/21.

**Animals.** *Mus musculus*, C57BL/6JRj male, 87 weeks of age ($\tilde{2}$0 months). Baseline body mass across all groups was 33.7 ± 2.6 g on Day 1, and 33.5 ± 2.9 g at study termination (Day 20). No significant differences in body weight were observed between treatment groups or over time. Animals were obtained from **JANVIER LABS** (CS 54105, Le Genest St Isle, 53941 Saint Berthevin Cedex, France) and housed under a 12:12 h light–dark cycle with ad libitum access to chow and water. All procedures were approved by the institutional animal care and use committee and conducted in accordance with institutional and national ethical guidelines. The study is reported in accordance with the ARRIVE guidelines.

#### Groups and regimens

Five arms were enrolled according to the interim dataset. Doses reflect the rationale to ensure an on-target pharmacodynamic effect with the MN route (higher payload vs IP) during this exploratory study. The overall study timeline, dosing schedule, and application workflow are summarized in Fig. 1. A concise summary of groups, routes, doses, and wear times is provided in Table 1.

**Table 1:** Study design summary: groups, sizes, route, dose and regimen.

Group (ID)	Route	Dose/admin	Regimen	Wear time	MN array	*n* (M/F)
Control (EG1)	–	–	No treatment	–	–	7 (**M**)
Daily IP (EG2)	IP	30 *μ*g	Daily, Days 1–20	n/a	n/a	7 (**M**)
Daily patch (EG4)	MN patch	$\tilde{0}$.4 mg/patch	Daily, Days 1–20	4 h/patch	19 MNs	7 (**M**)
Weekly patch (EG6)	MN patch	$\tilde{0}$.4 mg/patch	Weekly (Days 1, 7, 14, 21)	16 h/patch	19 MNs	9 (**M**)
Weekly IP (EG7)	IP	30 *μ*g	Weekly (Days 1, 7, 14, 21)	n/a	n/a	6 (**M**)

Rationale: A higher per-administration payload was used for the MN route to ensure sufficient tissue exposure in this proof-of-concept study; cumulative dose comparisons are not the focus here and will be addressed in future PK/PD work.

#### Measurements and endpoints

Grip strength was measured **daily on Days 1–20**. Terminal endpoints (Day 20/21) included dissected muscle mass (tibialis and soleus), histological fiber cross-sectional area distributions, and mitochondrial DNA content. MN performance metrics included residual drug in patch and drug-on-skin assays.

### Dosing

#### Dosing calendar (days 1–21)

The dosing regimen included both intraperitoneal (IP) injection and transdermal microneedle patch application groups. For the negative control group, mice received no treatment throughout the study. In the daily IP group, mice received a daily intraperitoneal injection of Pyr-Apelin-13 at a concentration of 0.5 *μ*mol/kg/day. In the daily patch group, mice received a daily application of a transdermal microneedle patch loaded with Pyr-Apelin-13. For the weekly IP group, mice received intraperitoneal injections of Pyr-Apelin-13 at 0.5 *μ*mol/kg on days 1, 7, 14, and 21. In the weekly patch group, mice received a transdermal microneedle patch once per week, specifically on days 1, 7, 14, and 21. The per-day nominal dosing schedule for all arms is detailed in Table 2.

**Table 2:** Nominal dose per day and group over the 21-day study. Dashes indicate no dosing event. Totals assume 30 *μ*g per IP administration and 0.4 mg per MN patch.

Day	Control	IP Daily [*μ*g]	MN Daily [mg]	MN weekly [mg]	IP Weekly [*μ*g]
1	–	30	0.4	0.4	30
2	–	30	0.4	–	–
3	–	30	0.4	–	–
4	–	30	0.4	–	–
5	–	30	0.4	–	–
6	–	30	0.4	–	–
7	–	30	0.4	0.4	30
8	–	30	0.4	–	–
9	–	30	0.4	–	–
10	–	30	0.4	–	–
11	–	30	0.4	–	–
12	–	30	0.4	–	–
13	–	30	0.4	–	–
14	–	30	0.4	0.4	30
15	–	30	0.4	–	–
16	–	30	0.4	–	–
17	–	30	0.4	–	–
18	–	30	0.4	–	–
19	–	30	0.4	–	–
20	–	30	0.4	–	–
21	–	–	–	0.4	30
**Total**	–	**600**	**8.0**	**1.6**	**120**

#### Residual drug analysis

Following application, all used microneedle patches were carefully removed from animals and stored in pre-labeled Petri dishes for subsequent evaluation of residual drug content and microneedle structural integrity. To assess potential surface loss, the exposed skin area was gently swabbed with a PBS-moistened cotton applicator. These swabs were transferred into microcentrifuge tubes, extracted in PBS, filtered through 0.22 *μ*m syringe filters, and analyzed by RP-HPLC. Swabs consistently showed negligible drug recovery, indicating minimal drug remained on the skin surface.

Used patches were subjected to in vitro release testing to quantify the amount of drug remaining after application. Each patch was incubated for 4 h in 1 mL PBS, followed by HPLC analysis of the filtrate. To establish the nominal drug load per patch, a parallel set of **reference patches** (unused patches from the same fabrication batch) underwent identical PBS release testing. These reference patches provided the baseline drug content (average *∼*0.386 mg per patch; Table 3).

The amount of Pyr-Apelin-13 delivered in vivo was calculated as:

1 $$\begin{aligned} \text {Delivered dose}&= (\text {Average reference patch load}) \nonumber \\&\quad - (\text {Residual drug in used patch}) \nonumber \\&\quad - (\text {Swabbed skin drug}) \end{aligned}$$

This approach yielded per-animal estimates of both the average dose delivered per application and the cumulative dose delivered over the study period. Results are summarized in Tables 4 and 5 for the daily and weekly dosing groups, respectively. On average, animals in the daily patch group received *∼*0.32 mg per application (cumulative *∼*6.5 mg across 20 applications), while animals in the weekly group received *∼*0.36 mg per application (cumulative *∼*1.44 mg across 4 applications).

This residual drug analysis confirms consistent drug loading across fabricated patches and demonstrates efficient in vivo release of Pyr-Apelin-13 with minimal loss on the skin surface. These results validate the robustness of the HeroPatch platform for reliable peptide delivery.

**Table 3:** Reference patches (unused) used to establish nominal Pyr-Apelin-13 loading.

Reference patch	In-vitro total drug release (mg)
Patch 1	0.332
Patch 2	0.394
Patch 3	0.365
Patch 4	0.389
Patch 5	0.382
Patch 6	0.391
Patch 7	0.376
Patch 8	0.387
Patch 9	0.383
Patch 10	0.380
Patch 11	0.392
Patch 12	0.381
Patch 13	0.386
Patch 14	0.382
Patch 15	0.384
Patch 16	0.390
**Average**	**0.38**

**Table 4:** Residual drug analysis in daily patch group (EG4).

Animal	Average drug dose delivered per patch (mg)	Total drug dose delivered (mg)
Animal 1	0.33	6.6
Animal 2	0.31	6.2
Animal 3	0.32	6.4
Animal 4	0.36	7.2
Animal 5	0.32	6.4
Animal 6	0.30	6.0
Animal 7	0.33	6.6

**Table 5:** Residual drug analysis in weekly patch group (EG6).

Animal	Average drug dose delivered per patch (mg)	Total drug dose delivered (mg)
Animal 1	0.35	1.40
Animal 2	0.36	1.44
Animal 3	0.34	1.36
Animal 4	0.37	1.48
Animal 5	0.36	1.44
Animal 6	0.38	1.52
Animal 7	0.35	1.40
Animal 8	0.37	1.48
Animal 9	0.36	1.44

### Patch application

Transdermal microneedle patches were applied using a standardized protocol to ensure consistent administration across animals. One day prior to the first application, mice were lightly shaved, and a depilatory cream was applied to remove residual hair. After hair removal, the skin was wiped thoroughly to remove excess cream, disinfected with 70% ethanol, and treated with a thin layer of moisturizing cream to prevent dryness.

On the day of application, mice assigned to the microneedle patch groups were placed under inhalational sevoflurane anesthesia to minimize handling stress and ensure stable positioning during patch placement. Anesthesia was induced with 6–8% sevoflurane until loss of the pedal reflex, and maintained at 3.5–4% for approximately 10 minutes, corresponding to the duration required for patch placement and initial adhesion. Control and intraperitoneal injection groups did not undergo inhalational anesthesia.

Before patch placement, the designated application site (behind the forelimbs or on the flanks) was disinfected with 70% ethanol. Patches were applied by gently stretching the skin to create a flat surface and securing the patch with slight pressure to ensure full microneedle engagement. Adhesion was reinforced using Smith & Nephew medical adhesive tape. Animals were individually housed after application, and patches were left in place for a fixed incubation period of 4 h.

At the end of the 4 h period, mice were briefly re-anesthetized with sevoflurane (3.5–4%) to facilitate careful removal of the patch. Microneedles were inspected post-removal to assess puncture marks and qualitative penetration efficiency. All mice recovered normally from anesthesia, and no adverse events related to patch administration or anesthetic exposure were observed.

### Functional testing

Neuromuscular function was evaluated using a forelimb grip strength test, which is widely employed to assess motor performance and potential treatment-related effects. Grip strength was measured daily with a digital grip strength dynamometer equipped with a horizontal metal grid. The device was calibrated prior to each testing session to ensure accuracy and reproducibility. Each mouse was gently lifted by the tail and allowed to grasp the metal grid with its forepaws. The animal was then pulled backward along the horizontal axis with constant force until it released its grip, and the peak tension force (in grams) was automatically recorded by the device. For each mouse, five consecutive trials were performed per session with an inter-trial interval of at least 10 s to minimize fatigue. To account for inter-animal variability, grip strength values were normalized to the body weight recorded on the day of testing and expressed as percentage grip strength (g/g body weight *×* 100). Body weight was monitored throughout the study to detect any treatment-related changes in general health. Trials in which the mouse failed to grasp the grid with both forepaws or released prematurely without measurable resistance were excluded from analysis. Animals showing signs of distress, injury, or abnormal behavior were not subjected to testing on that day and were re-evaluated at the next scheduled session.

### Tissue collection & muscle mass

At the end of the study (days 20–21), mice were euthanized using inhalational sevoflurane (6–8%) until deep anesthesia was achieved and loss of reflexes was confirmed, followed by cervical spine dislocation, in accordance with approved ethical protocols. The tibialis anterior and soleus muscles were selected for analysis because they represent distinct skeletal muscle phenotypes: the tibialis anterior is predominantly fast-twitch and sensitive to structural remodeling, whereas the soleus contains a higher proportion of slow-twitch, oxidative fibers commonly used to assess metabolic adaptations.

Following euthanasia, both muscles were carefully dissected from each hindlimb. All visible connective tissue and tendon remnants were removed with fine forceps to ensure precise isolation. Each muscle was briefly blotted on filter paper to remove surface moisture and immediately weighed on a calibrated analytical balance. Muscle masses were recorded in milligrams and normalized to body weight (mg/g body weight) to account for inter-animal variability.

After weighing, tissues were either snap-frozen in liquid nitrogen for molecular analysis or placed in histology cassettes and fixed in 4% paraformaldehyde for subsequent sectioning and histological evaluation.

### Histology & CSA quantification

Dissected skeletal muscles designated for histological analysis were fixed in freshly prepared 4% paraformaldehyde (PFA) in phosphate-buffered saline (PBS) and stored at 4 $^{\circ }$C until processing. After fixation, samples were dehydrated using an Automatic Benchtop Tissue Processor (Leica TP1020), paraffin embedded with a HistoCore Arcadia H (Leica, Cat. No. 14039357258), and sectioned parallel to the sagittal plane at 5 *μ*m thickness using a Leica RM2125 RTS automated rotary microtome. Sections were mounted onto StatLab KT5+ advanced adhesive microscope slides to ensure secure adherence during histological processing.

Mounted tibialis anterior and soleus sections were stained with hematoxylin and eosin (H&E; Hematoxylin Harris, VWR 95057-858; Eosin, VWR 10143-132). Hematoxylin labeled nuclear material in blue–purple, and eosin counterstained cytoplasmic and extracellular matrix components in pink, enabling high-contrast visualization of myofiber architecture.

All slides were imaged under brightfield illumination at 20*×* magnification using a Leica DM LS2 microscope equipped with a Leica DFC500 digital camera. Exposure time, white balance, and gain settings were kept constant across samples to ensure comparability. Muscle fiber cross-sectional area (CSA) was quantified using ImageJ/FIJI following a semi-automated pipeline: images were converted to 8-bit, thresholded, and individual fibers were outlined. CSA values were extracted from at least 400 fibers per muscle to ensure representative sampling.

To evaluate shifts in fiber size distribution across treatment groups, CSA values were categorized into predefined numerical ranges: *<800~μ*m$^{2}$, *800-1500~μ*m$^{2}$, and *>1500~μ*m$^{2}$. These bins were used descriptively to assess the relative proportion of smaller, intermediate, and larger fibers within each muscle. Identical bin ranges were applied to both muscles to maintain analytical consistency when comparing treatment-related distribution shifts within each muscle type.

### Mitochondrial DNA analysis

Total DNA was extracted from snap-frozen skeletal muscle samples using the KingFisher Apex automated purification instrument (Thermo Fisher Scientific, USA) according to the manufacturer’s instructions. DNA concentration and purity were determined spectrophotometrically, and only samples with an A$_{260}$/A$_{280}$ ratio between 1.8 and 2.0 were included for downstream analysis.

Relative mitochondrial DNA (mtDNA) abundance was quantified by real-time PCR using the KAPA SYBR FAST qPCR Kit (KK4608, KAPA Biosystems) on a QuantStudio 5 Flex Real-Time PCR System (Thermo Fisher Scientific, USA). Two mitochondrial-encoded genes, *mt-ND1* (NADH dehydrogenase subunit 1) and *mt-Co1* (cytochrome c oxidase subunit 1), were amplified as markers of mtDNA copy number. The nuclear gene *Ppia* (Peptidylprolyl isomerase A) was amplified in parallel for normalization, as its expression is stable in skeletal muscle under aging and treatment conditions. This assay quantifies relative mtDNA abundance and is not a direct measurement of mitochondrial biogenesis.

Primer sequences are listed in Table 6.

**Table 6:** Primer sequences used for mitochondrial DNA analysis.

Target gene	Forward (5’–3’)	Reverse (5’–3’)
*Ppia* (nuclear reference)	GGGGTTCTTGCTTCAGTGTTC	TCAGCAACCAGGGGTAGATCA
*mt-Co1*	TCTTTGATCCCGCTGGAGG	TCTGGGTGCCCAAAGAATCAG
*mt-ND1*	GCATCTTATCCACGCTTCCG	ACTCCCGCTGTAAAAATTGGT

Thermal cycling conditions were as follows:

- Pre-incubation: 95 $^{\circ }$C for 3 min
- Amplification (40 cycles): 95 $^{\circ }$C for 3 s, 60 $^{\circ }$C for 20 s, 72 $^{\circ }$C for 30 s
- Melt curve: 95 $^{\circ }$C for 15 s, 55 $^{\circ }$C for 1 min, 95 $^{\circ }$C for 15 s

All reactions were performed in triplicate. Relative mtDNA abundance was calculated using the $\Delta \Delta C_{t}$ method and expressed as the ratio of mitochondrial to nuclear DNA signals.

### Microneedle patch fabrication

Microneedle (MN) patches were fabricated to deliver Pyr-Apelin-13 in a solid-state formulation. The matrix formulation consisted of a biocompatible resin component, Pyr-Apelin-13 in powder form, Tween-80, and sodium phosphate in a ratio of 6:6:1:1 (w/w). The drug–excipient mixture was placed into silicone molds and cured under UV light for 60 min to obtain solidified microneedle arrays. Each patch consisted of an array containing 19 microneedles with dimensions of 1.1 mm in height and 0.2 mm at the base diameter.

### HPLC method and drug release quantification

Drug quantification was performed by reverse-phase HPLC on a Agilent 1260 Infinity II HPLC system using a C18 column (5 *μ*m) at room temperature. Mobile phase A was 0.1% Trifluoroacetic acid (TFA) in water and mobile phase B was 0.1% TFA in acetonitrile, with a gradient from 81:19 (A:B) at 0 min to 71:29 at 20 min. The flow rate was 1.0 mL/min, detection wavelength 220 nm, and injection volume 20 *μ*L.

For cumulative release studies, microneedle patches were incubated in PBS, and 1 mL aliquots of the medium were collected at 30 min, 1 h, 2 h, 3 h, 4 h, and 24 h. Samples were filtered (0.22 *μ*m) and analyzed by HPLC, with concentrations determined from a standard calibration curve.

### Drug stability in the microneedle patch

Drug stability was assessed to evaluate the integrity of Pyr-Apelin-13 within the microneedle patch formulation and following release from the patches. Fabricated microneedle patches were placed in phosphate-buffered saline (PBS) for a total of 4 h to achieve in vitro release. Aliquots of 1 mL release medium were collected and analyzed using reverse-phase high-performance liquid chromatography (HPLC) as described previously. For long-term stability testing, microneedle patches were stored either at room temperature or at 4 $^{\circ }$C in a refrigerator. After one month, the patches were subjected to in vitro release in PBS, and the collected samples were analyzed by HPLC to detect any potential degradation or aggregation of Pyr-Apelin-13.

### Statistics

**Statistics and presentation.** Unless noted, tests were two tailed. Assumptions were checked by Shapiro–Wilk tests and inspection of residuals; Welch’s tests were used where variances differed; *p* values for panel (b) were multiplicity-adjusted by Holm. In panel (a), boxplots depict median and interquartile range (IQR) with whiskers at 1.5 *×* IQR and points as individual animals. In panel (c), points are group means with SD bars; lines are ordinary least-squares fits. Significance codes: ns *p≥ 0.05*, * *p<0.05*, ** *p<0.01*, *** *p<0.001*, **** *p<0.0001*. Sample sizes were *n* per group: Control $=\,\langle n_c\rangle$, Daily patch $=\,\langle n_{dp}\rangle$, Daily IP $=\,\langle n_{di}\rangle$, Weekly patch $=\,\langle n_{wp}\rangle$, Weekly IP $=\,\langle n_{wi}\rangle$ (replace with actual *n*).

## Results

### Microneedle design, validation and application

HeroPatch patches used in this study consist of a hexagonal array (active area *≈*9 mm) containing 19 conical MNs (nominal height *≈*1.1 mm). *In vitro* release testing of the formulation selected for in vivo studies showed sustained release of Pyr-Apelin-13 over a 4-hour window with near-complete recovery (100% release) by 4 h (mean ± SD). HPLC comparison of patch extracts at day 0 and after 1 month storage at 4 °C yielded identical retention times to the reference API with no detectable degradants, indicating refrigerated stability. Optical imaging and SEM demonstrated intact, smooth MNs prior to application and a transition to a whitish, porous microstructure after release consistent with payload depletion and porosity development in the polymer matrix. In mice, patches were applied to shaved, stretched dorsal skin by manual bilateral pinch/press (10–30 s); removal revealed a puncture pattern with no gross erythema under these conditions (see Fig. 2c–m).

To establish the feasibility of delivering Pyr-Apelin-13 using our solid-state microneedle platform, we first characterized the patch structure, geometry, and application in mice. Figure 2 illustrates the patch design and usage workflow: the patch consists of an adhesive ring and a central microneedle (MN) array containing 19 MNs in a 9-mm diameter area. A magnified view shows the resin-based solid microneedles prior to application. The patch was applied manually to the shaved dorsal skin of mice, and puncture sites were clearly visible after removal. Post-application, the microneedles appeared white due to drug release and exposure of the porous resin structure.

### Functional outcomes (grip strength trajectories)

Forelimb grip strength was quantified for 20 days across five arms: Control, Daily Apelin-13 by microneedle patch (Daily patch), Daily Apelin-13 by intraperitoneal injection (Daily IP), Weekly patch, and Weekly IP. To reduce inter-animal variability and account for age-related differences in body size, all measurements were normalized to each animal’s body weight and reported as grams of force per gram of body weight (g/g). Grip strength reflects forelimb function and therefore provides a complementary readout to the hindlimb muscles analyzed histologically.

**Fig. 3:**
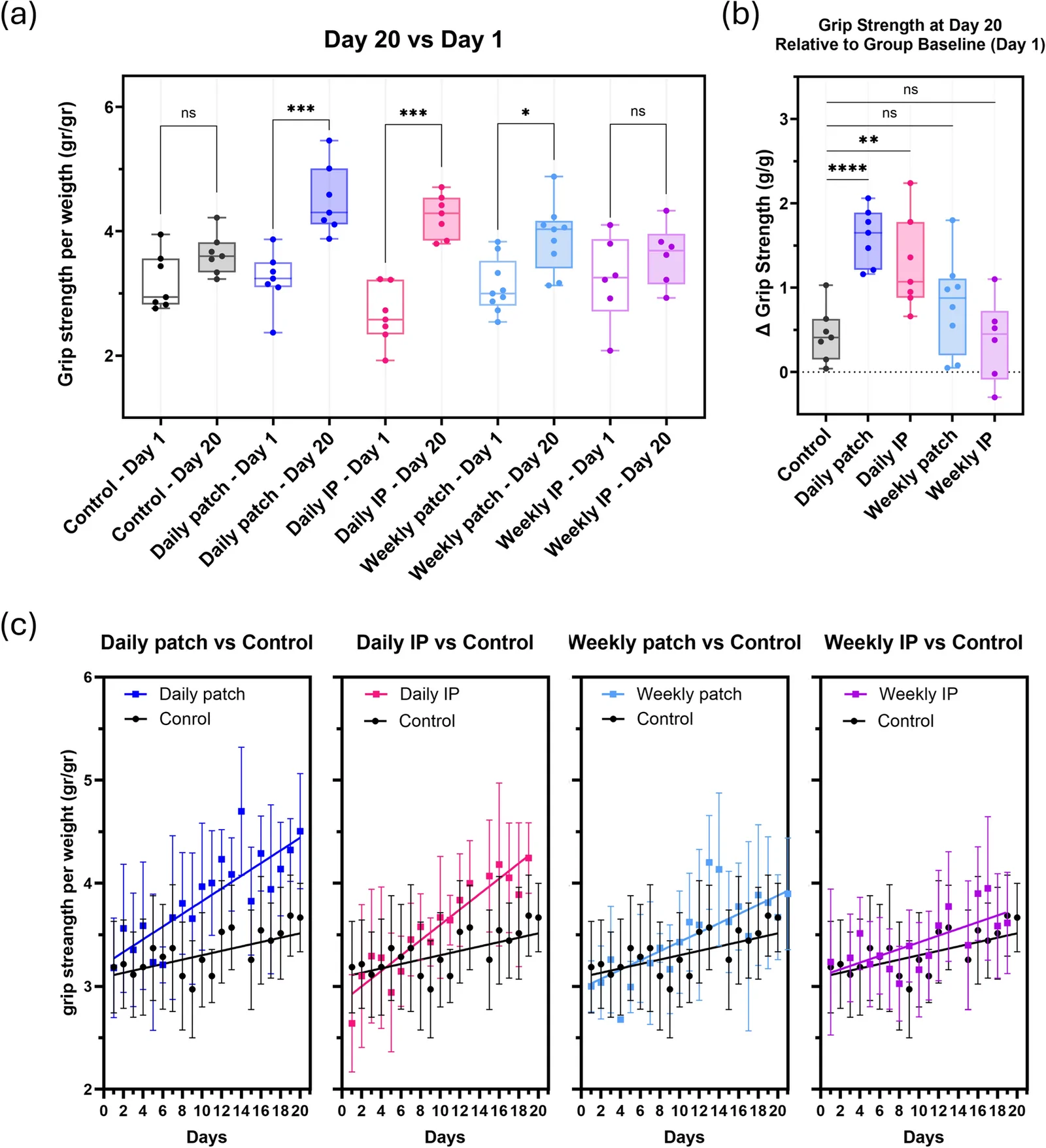
Apelin-13 improves forelimb grip strength, with the largest gains observed under daily microneedle patch dosing. **(a)** Within-group comparison of normalized grip strength (g/g) at Day 1 vs Day 20. Daily patch and Daily IP show significant gains (***), Weekly patch shows a smaller but significant increase (*), and Weekly IP and Control show no change (ns). **(b)** Baseline-adjusted improvement at Day 20, calculated as *Δ*Grip *=* Day 20 minus group Day 1 mean. Daily patch shows the largest improvement versus Control (****), Daily IP is also significantly elevated (**), while weekly regimens are not different from Control (ns). **(c)** Longitudinal trajectories (group mean ±SD) with ordinary least-squares trend lines. Daily regimens show steeper increases than Control; weekly regimens are intermediate. *Note:* Weekly groups received 4 total administrations, compared to 20 for daily groups, resulting in substantially lower cumulative exposure. Statistics: paired two-tailed *t*-tests for panel (**a**); Welch’s two-tailed *t*-tests vs Control with Holm correction for panel (**b**). Significance codes: ns *p≥ 0.05*, * *p<0.05*, ** *p<0.01*, *** *p<0.001*, **** *p<0.0001*.

Figure 3 summarizes three complementary analyses designed to (i) test within-group improvement from baseline, (ii) compare end-of-study performance across groups with baseline adjustment, and (iii) visualize longitudinal trajectories and their rates of change.

**Within-group change from baseline (Fig.** 3**a).** For each arm, Day 1 values were compared to Day 20 values using paired, two-tailed *t*-tests. Daily patch and Daily IP exhibited large, statistically significant gains from baseline (***), Weekly patch showed a smaller but significant increase (*), and Weekly IP did not differ from its baseline (ns). Control remained unchanged (ns). These results indicate that Apelin-13 improves normalized grip strength over 20 days under the dosing conditions tested.

**Between-group difference at study end with baseline adjustment (Fig.** 3**b).** To compare treatment arms at the end of the study while accounting for starting strength, we computed a baseline-adjusted change for each mouse, $\Delta \textrm{Grip} = \textrm{Grip}_{\text {Day 20}} - \overline{\textrm{Grip}}_{\text {group, Day 1}}$. We then contrasted each treatment to Control at Day 20 using Welch’s two-tailed *t*-tests with Holm correction for multiple comparisons. Daily patch displayed the largest improvement versus Control (****), Daily IP was also significantly higher (**), and weekly regimens were not different from Control (ns). These findings corroborate the within-group effects and show that the greatest improvements were observed under daily dosing.

**Trajectory and rate of improvement (Fig.** 3**c).** Longitudinal plots of group means (±SD) across the 20-day study show steadily rising trajectories for Daily patch and Daily IP, with visibly steeper ordinary least-squares trend lines than Control; Weekly patch is intermediate and Weekly IP tracks close to Control. Although panel (c) is presented descriptively, this pattern is consistent with stronger cumulative Apelin-13 exposure under daily dosing. Formal inference on slope differences could be performed via ANCOVA or a mixed-effects model with a group*×*day interaction; visually, the ordering of slopes mirrors the significance pattern in panels (a)–(b). Because only patch groups underwent brief anesthesia for patch placement, mild handling or arousal differences cannot be fully excluded as contributors to day-to-day variability.

**Interpretation and dose considerations.** Across the three complementary views, Apelin-13 increases forelimb grip strength normalized to body weight. Apparent group ordering (Daily patch *≳* Daily IP > Weekly patch *≳* Weekly IP *≈* Control) should be interpreted with caution because the weekly arms received only four administrations over the study versus twenty for the daily arms (i.e., *∼*5*×* fewer), resulting in markedly lower cumulative dose and exposure. Thus, the smaller gains seen with weekly delivery reflect underexposure and do not isolate schedule effects. With exposure-matched regimens—e.g., increasing weekly per-dose amounts to match the 20-day cumulative dose of the daily schedule, or performing PK-guided AUC matching—the contributions of schedule versus dose can be clarified.

Route may also influence exposure profiles: microneedle patches are expected to produce smoother and more sustained delivery, whereas IP delivers short bolus peaks. These pharmacokinetic differences could contribute to the relative performance of the patch under the conditions tested, but cannot be disentangled without direct PK measurement. Potential practice effects from repeated testing were limited by the relatively flat control trajectory. Overall, the data demonstrate Apelin-13 efficacy and nominate the daily patch as the most effective regimen under the tested doses, motivating future exposure-matched studies to determine whether less frequent dosing can yield comparable benefit.

### Skeletal muscle mass

**Fig. 4:**
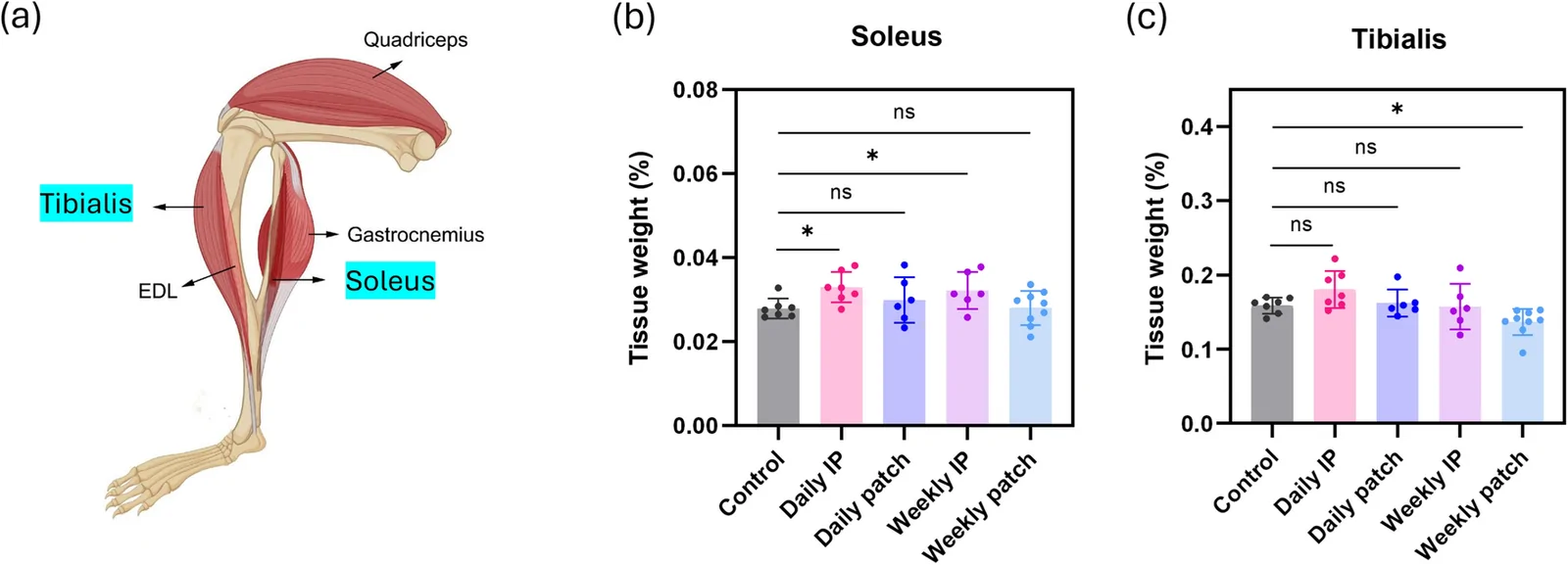
Muscle weight analysis after 21 days of Apelin-13 treatment. (**a**) Anatomical illustration of tibialis anterior (TA), soleus, gastrocnemius, and EDL muscles in the mouse hind limb, indicating tissues collected for analysis. (**b**) Normalized soleus weight (percentage of body weight). Daily and weekly IP groups showed modest but significant increases compared with Control, while patch groups did not differ significantly from Control. (**c**) Normalized tibialis anterior weight. No significant differences were detected except for a small reduction in the weekly patch group relative to Control. Data are presented as mean ± SD. Statistical significance: * *p<0.05*, ns = not significant.

To complement the functional (grip strength) and histological (fiber CSA) outcomes, we quantified the wet weight of the soleus and tibialis anterior muscles at study termination and normalized these values to body weight (Fig. 4). Panel (a) illustrates the anatomical location of the dissected muscles.

In the soleus (Fig. 4b), daily and weekly microneedle patch groups showed no significant difference in normalized tissue weight relative to untreated controls. In contrast, both daily and weekly intraperitoneal (IP) groups exhibited modest but statistically significant increases.

In the tibialis anterior (Fig. 4c), there were no significant differences among most groups, with the exception of a small reduction in the weekly patch group compared to control.

Taken together, these findings indicate that patch delivery did not measurably alter gross muscle mass, despite producing clear improvements in functional (grip strength) and microscopic (CSA) endpoints. This discrepancy likely reflects the greater sensitivity of functional and histological measures compared to bulk muscle weight, which may also be influenced by technical variability during dissection.

### Muscle fiber morphology

**Fig. 5:**
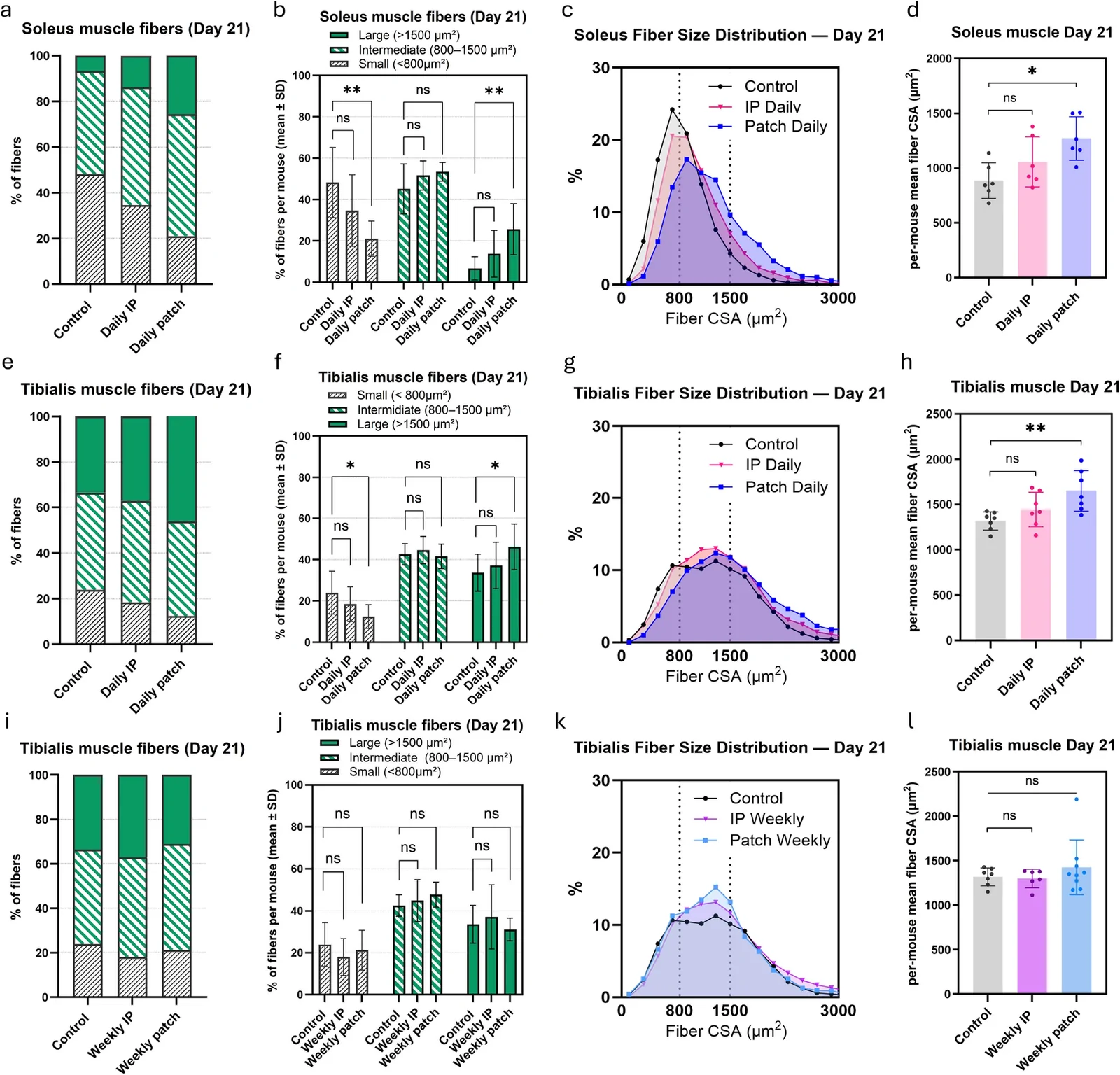
Fiber-size distributions and cross-sectional area (CSA) after 21 days of Apelin-13 treatment. Fiber CSA values were grouped into numerical bins ($<800~\mu \textrm{m}^2$, 800–1500 $\mu \textrm{m}^2$, $>1500~\mu \textrm{m}^2$) to visualize per-mouse distributions of smaller versus larger fibers. (a–d) Soleus muscle, daily regimens: daily intraperitoneal (IP) and daily patch treatment both reduce the fraction of smaller fibers and increase the fraction of larger fibers, with right-shifted fiber-size distributions and higher mean CSA. (e–h) Tibialis muscle, daily regimens: similar rightward shifts and increased mean CSA are observed, most prominently with daily patch; daily IP shows comparable but slightly smaller effects. (i–l) Tibialis muscle, weekly regimens: weekly IP and weekly patch dosing do not produce significant shifts in fiber-size distribution or mean CSA, consistent with lower cumulative exposure. Data represent numerical CSA distributions and are not diagnostic categories; bars denote mean ± SD. Statistical comparisons used one-way ANOVA with post hoc tests; ns = not significant, * *p<0.05*, ** *p<0.01*.

To evaluate treatment-associated changes in skeletal muscle structure, we analyzed fiber-size distributions and mean cross-sectional area (CSA) in soleus and tibialis muscles after 21 days of treatment (Fig. 5). CSA values were grouped into predefined numerical bins to visualize shifts in the distribution of smaller versus larger fibers, and mean CSA was computed on a per-mouse basis to avoid sampling bias. These structural findings parallel the functional improvements observed in grip strength.

**Soleus muscle.** Daily Pyr-Apelin-13 patch treatment produced a clear rightward shift in the CSA distribution, reflected by fewer fibers in the smallest-size bin and a corresponding increase in the frequency of larger fibers. Daily IP treatment showed a similar pattern. These distributional changes were accompanied by significantly increased mean CSA in the daily patch group compared with control, indicating enlargement of average fiber size under daily dosing.

**Tibialis muscle.** A comparable rightward shift in CSA distribution was observed in tibialis muscle with daily patch treatment, again characterized by a reduced proportion of the smallest fibers and a higher proportion of larger fibers. Mean CSA was significantly increased relative to control. Daily IP treatment displayed similar trends, supporting a consistent Apelin-13–associated increase in fiber size across hindlimb muscles.

**Weekly treatment.** Weekly patch administration did not produce statistically detectable shifts in CSA distribution or mean CSA relative to control, although a modest, non-significant reduction in the smallest fibers was noted. The absence of measurable structural changes under weekly dosing is consistent with its substantially lower cumulative exposure over the 21-day period.

**Fig. 6:**
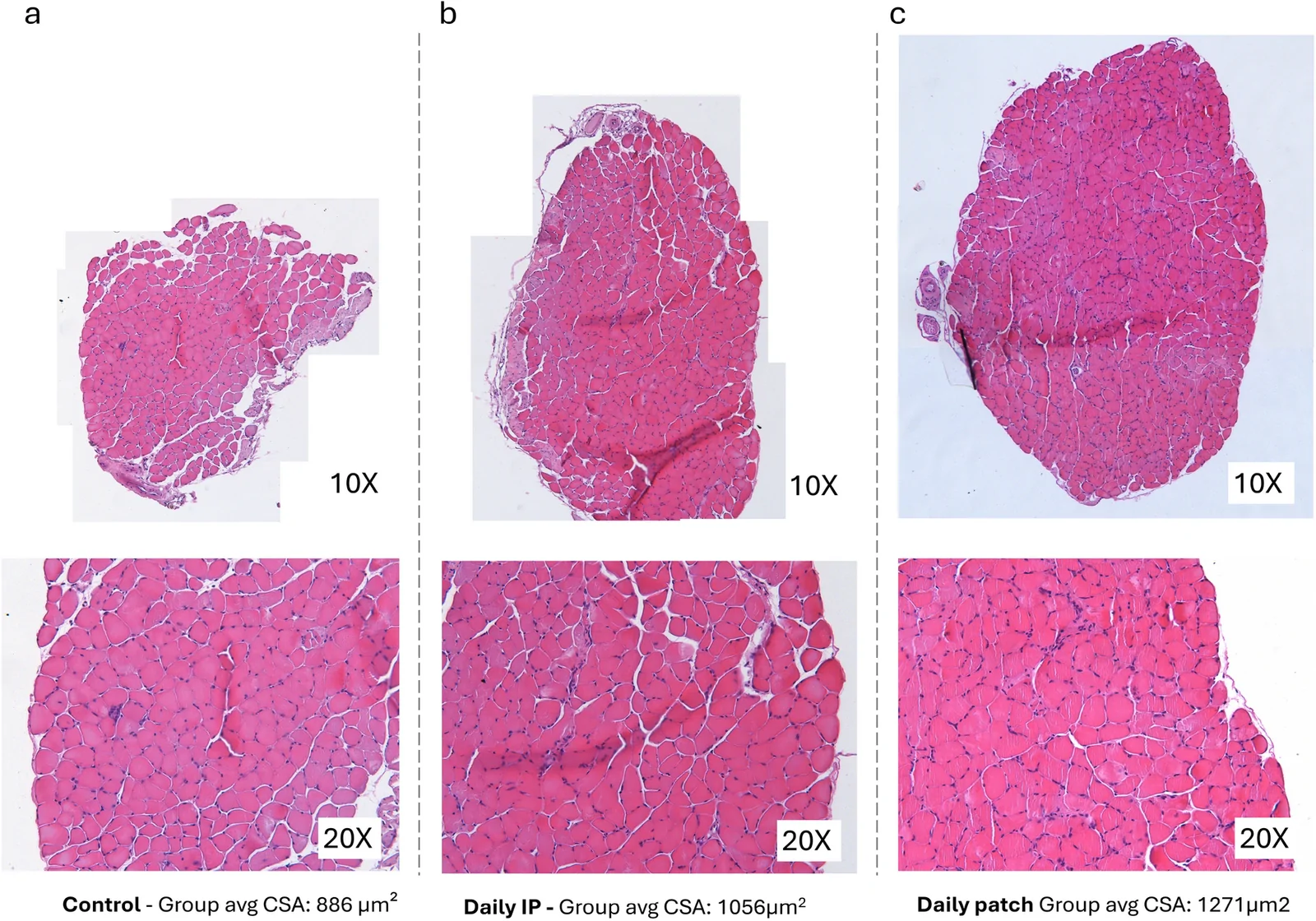
Representative H &E-stained tibialis anterior cross-sections. Sections were selected from animals whose image-level mean CSA was close to the group mean, providing representative visualization of fiber-size differences. **Control**: animal 1 (image mean CSA = 843 *μ*m$^{2}$; group mean = 886 *μ*m$^{2}$). **Daily IP**: animal 4 (image mean = 922 *μ*m$^{2}$; group mean = 1056 *μ*m$^{2}$). **Daily Patch**: animal 1 (image mean = 1180 *μ*m$^{2}$; group mean = 1271 *μ*m$^{2}$). Daily Apelin-13 administration, particularly via the microneedle patch, is associated with visibly larger myofibers compared with Control under the conditions tested.

Overall, these findings show that *daily delivery of Pyr-Apelin-13, particularly via the microneedle patch, is associated with a shift toward larger muscle fibers and increased mean CSA* in both soleus and tibialis muscles. Because the CSA bins are numerical rather than diagnostic, these observations should be interpreted as distributional shifts rather than categorical classifications of fiber state. Representative H&E images illustrating these CSA differences are shown in Fig. 6.

#### Muscle fiber quantification

For histological analysis, muscle fiber cross-sectional area (CSA) was quantified from transverse sections of the tibialis anterior and soleus muscles. Fiber segmentation and CSA measurements were performed using representative non-overlapping fields selected across the entire muscle cross-section for each animal.

The total number of muscle fibers quantified per animal varied depending on muscle type and tissue availability. For the tibialis anterior muscle, fiber counts were obtained for all treatment groups, including control, daily intraperitoneal (IP), daily microneedle patch, weekly microneedle patch, and weekly IP groups. For the soleus muscle, fiber quantification was performed for the control, daily IP, and daily microneedle patch groups. Weekly dosing groups were not analyzed for soleus muscle histology.

Detailed per-animal fiber counts for each muscle and treatment group are provided in Tables 7 and 8.

**Table 7:** Number of muscle fibers quantified per animal for tibialis anterior muscle CSA analysis.

Animal	EG1 control	EG2 daily IP	EG4 daily patch	EG6 weekly patch	EG7 weekly IP
Animal 1	881	1067	529	985	642
Animal 2	1075	725	871	671	958
Animal 3	995	761	728	372	739
Animal 4	876	985	584	934	953
Animal 5	707	790	533	762	733
Animal 6	1396	693	491	489	768
Animal 7	969	669	506	663	–
Animal 8	–	–	–	623	–
Animal 9	–	–	–	749	–

**Table 8:** Number of muscle fibers quantified per animal for soleus muscle CSA analysis.

Animal	EG1 Control	EG2 Daily IP	EG4 Daily Patch
Animal 1	1146	925	1064
Animal 2	1071	459	936
Animal 3	493	789	655
Animal 4	476	657	565
Animal 5	752	1196	837
Animal 6	852	1159	427
Animal 7	442	844	981

### Mitochondrial content

We quantified mitochondrial DNA (mtDNA) content in **soleus** and **tibialis** muscles by qPCR on genomic DNA using two mitochondrial targets, *mt-Co1* and *mt-Nd1*, normalized to the nuclear gene *Ppia*. For each plate, per-mouse values were computed as $\Delta C_t = C_t(\text {mt}) - C_t(\text {Ppia})$, referenced to the plate’s control mean to obtain $\Delta \Delta C_t$, and expressed as fold change $2^{-\Delta \Delta C_t}$ (Control = 1). Per-mouse RQ values from two independent plates were pooled for analysis.

**Fig. 7:**
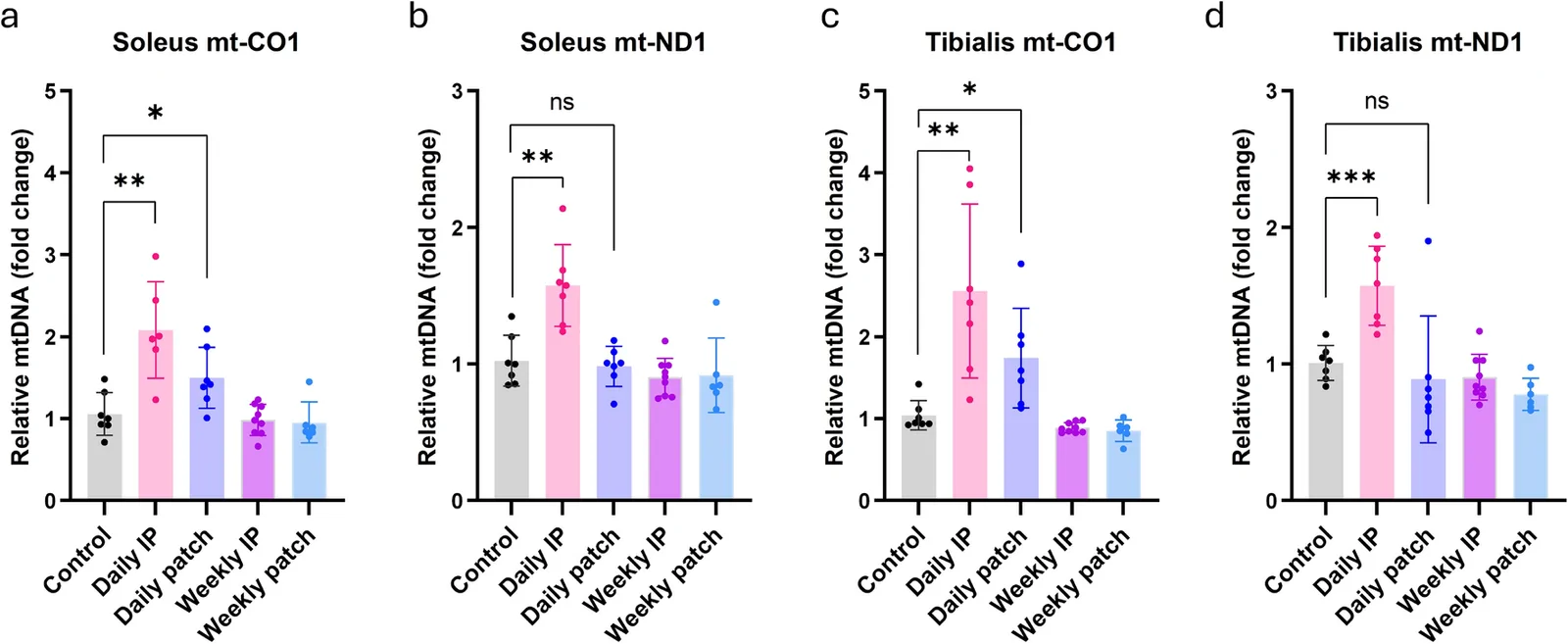
Relative mitochondrial DNA content in skeletal muscle. (**a**) Soleus *mt-Co1*; (**b**) Soleus *mt-Nd1*; (**c**) Tibialis *mt-Co1*; (**d**) Tibialis *mt-Nd1*. Mitochondrial gene targets were normalized to nuclear *Ppia* and expressed as $2^{-\Delta \Delta C_t}$ relative to Control (set to 1). Bars show mean ± SEM with individual mouse values overlaid. Two-tailed unpaired *t*-tests were used for comparisons versus Control; ns *p≥ 0.05*, * *p<0.05*, ** *p<0.01*, *** *p<0.001*. Daily IP treatment demonstrated the most pronounced increases in mtDNA markers, while Daily Patch produced moderate but statistically significant elevations for *mt-Co1*. Weekly regimens did not differ from Control.

Daily intraperitoneal Apelin-13 (**Daily IP**) produced the largest increases in mtDNA markers across both muscles and both targets (Fig. 7a–d). Daily transdermal delivery via HeroPatch (**Daily Patch**) resulted in moderate increases that reached statistical significance for *mt-Co1* in both muscles, with *mt-Nd1* showing a similar but non-significant trend. Weekly regimens (**Weekly IP** and **Weekly Patch**) did not differ from Control. These findings are consistent with a dose- and exposure-dependent mitochondrial response, in which greater cumulative Apelin-13 delivery under the daily schedules is associated with larger increases in mtDNA markers. Because pharmacokinetic profiles were not measured in this study, differences between delivery routes should be interpreted cautiously and considered descriptive rather than mechanistic.

*Statistics.* Bars show mean ± SEM with individual mice overlaid (*n=7*–9 per group). Two-tailed unpaired *t*-tests were performed for each treatment versus Control; exact *p*-values are annotated in the panels (*p<0.05, **p<0.01, ***p<0.001; ns, not significant).

### Delivery efficiency and microneedle performance

In vitro release testing of Pyr-Apelin-13 from reference patches demonstrated complete release within 4 h (100% drug recovery). The nominal patch loading, determined from reference patches, was *0.38 ± 0.01* mg (Table 3).

Residual drug analysis from in vivo studies revealed that the average dose delivered per application was 0.32 mg in the daily dosing group (Table 4) and 0.36 mg in the weekly dosing group (Table 5). These values closely match the nominal loading determined in vitro, corresponding to a delivery efficiency of *∼*83% in the daily group and *∼*93% in the weekly group.

Drug recovery from skin swabs was negligible, consistently *<1%* of the nominal dose, indicating minimal drug loss at the skin surface. The slight variability observed across animals is attributed to biological variation in skin penetration and application technique, rather than limitations of the microneedle platform.

Taken together, these results demonstrate that the in vivo delivery performance of the microneedle patch is highly consistent with in vitro release kinetics. The close correspondence between nominal patch loading and delivered dose confirms both the reproducibility of patch fabrication and the efficiency of drug release in vivo. The HeroPatch platform thus provides robust and predictable peptide delivery with minimal residual loss.

## Discussion

Daily transdermal delivery of Pyr-Apelin-13 using HeroPatch improved both functional performance and muscle fiber morphology in aged mice. These findings are directionally consistent with prior reports that apelin enhances mitochondrial function and muscle regeneration in the context of aging^1^. The present study extends this work by demonstrating that a non-invasive microneedle (MN) patch can reproduce many of the beneficial outcomes previously associated with daily intraperitoneal administration.

Across functional, morphological, and mitochondrial readouts, daily dosing produced the clearest effects, whereas weekly schedules—delivering substantially lower cumulative exposure—showed little or no measurable improvement. This pattern suggests that overall exposure plays an important role in determining the magnitude of the response. Daily MN delivery achieved intermediate increases in mtDNA markers relative to daily injection, indicating that the patch can engage mitochondrial pathways, although the degree of engagement may depend on relative exposure levels and delivery kinetics. Because pharmacokinetic data were not collected, differences between routes should be interpreted descriptively rather than mechanistically.

The consistent shift toward larger muscle fibers and the increase in mean CSA under daily regimens align with the observed improvements in grip strength, suggesting a coordinated functional and structural adaptation. The mtDNA results, although modest for the Daily Patch group, showed concordant trends across *mt-Co1* and *mt-Nd1*, supporting the interpretation that Apelin-13 can stimulate mitochondrial remodeling through MN delivery. However, the precise relationship between dosing schedule, exposure profile, and mitochondrial response remains to be defined.

Importantly, the MN patches were well tolerated in aged mice, despite age-associated changes in skin thickness and elasticity. The robustness of application and the reproducibility of biological effects highlight the potential of MN delivery for targeting age-related muscle decline with reduced invasiveness compared to daily injections. This feature is particularly relevant for chronic therapies in older populations, where adherence, comfort, and ease of administration are major considerations.

Future studies should include direct pharmacokinetic characterization of Apelin-13 delivered via MN patches to establish exposure–response relationships and to clarify the relative contributions of cumulative dose versus delivery kinetics. Evaluating extended-wear or higher-loading patch formats may further enhance exposure while preserving ease of use. Finally, testing in larger animal models with skin architecture closer to humans will be essential for translation toward clinical applications.

## Limitations

This study has several limitations. First, weekly and daily regimens were not dose-matched, which limits the ability to distinguish scheduling effects from differences in cumulative exposure. Second, although the sample sizes (n = 6–9 per group) were sufficient to detect differences in CSA and grip strength, larger cohorts would improve precision and allow for more robust modeling of inter-individual variability. Third, pharmacokinetic data were not collected, preventing direct comparison of exposure profiles between intraperitoneal and microneedle delivery. As a result, differences between routes should be interpreted descriptively. Future work will incorporate exposure-matched dosing, expanded group sizes, and optimized PK methods to better resolve the exposure–response relationship for Apelin-13 delivered via the HeroPatch platform.

## Perspective

While daily application showed the strongest effects under the conditions tested, opportunities remain to optimize the formulation and delivery profile of the HeroPatch. Engineering longer-wear or sustained-release designs may enable reduced application frequency while maintaining adequate exposure. Future iterations could also explore co-delivery of complementary peptides or metabolic modulators, which may enhance functional and mitochondrial outcomes. More broadly, these findings illustrate the potential of microneedle-based transdermal systems as an alternative to injection for the chronic delivery of short-acting peptides, and motivate further development toward clinically scalable regenerative therapies.

## Conclusion

Apelin has been widely studied for its roles in muscle physiology, mitochondrial regulation, and metabolic homeostasis, yet its therapeutic development has been hindered by rapid systemic clearance and the inconvenience of repeated injections. The present study demonstrates that a microneedle-based transdermal system can deliver Pyr-Apelin-13 in a manner that produces measurable improvements in muscle function, fiber morphology, and mitochondrial markers in aged mice. These findings show that daily microneedle delivery can engage biological pathways previously associated with injected Apelin-13, supporting the feasibility of a non-invasive approach for chronic peptide administration.

Although daily microneedle dosing produced effects that were directionally similar to daily intraperitoneal administration, the relative contributions of cumulative dose, delivery kinetics, and exposure profile remain to be defined. As such, the present results should be viewed as evidence of transdermal activity rather than equivalence between routes. The solid-state format of the HeroPatch platform, which can accommodate milligram-level peptide loading, suggests the potential for scaling toward higher exposures or longer-wear formats in future studies. This strategy may extend to additional short half-life peptides for which delivery challenges have limited therapeutic exploration.

Overall, the data support microneedle-based delivery as a promising avenue for advancing peptide therapeutics in aging and muscle health. Further work incorporating pharmacokinetics, dose matching, and larger-animal testing will be essential for translating these findings toward clinical applications.

## Data Availability

The datasets generated and analyzed during the current study are available from the corresponding author on reasonable request. All raw and processed data supporting the findings of this study are archived at Anodyne Nanotech and its collaborating facilities.
